# Supplementary material for: Lived experience of people on anti-retro viral therapy in the context of covid-19: A phenomenological study
Source: PLoS One. 2023 Nov 2;18(11):e0286292. doi: 10.1371/journal.pone.0286292 (PMC10621913; doi:10.1371/journal.pone.0286292)
Supplement: S1 File — (RTF) [file pone.0286292.s001.rtf]

List of all objects
______________________________________________________________________

HU:	tadele edited atlas
File:	 [C:\Users\HAB-TECH\Desktop\tadele edited atlas.hpr7]
Edited by:	Super
Date/Time:	2021-06-15 12:14:50
______________________________________________________________________


HU
______________________________________________________________________

tadele edited atlas

Primary Documents
______________________________________________________________________

P 6: par.0001.docx {33}
P 9: parti-0003.docx {20}
P10: parti-0004.docx {29}
P11: parti-0005.docx {43}
P12: parti-0006.docx {25}
P16: parti-00012.docx {53}
P19: parti-0009.docx {36}
P20: parti-00013.docx {45}
P21: parti-2m.docx {26}
P22: parti-10.docx {35}
P23: parti-00011.docx {30}
P24: parti-00014.docx {36}
P26: parti-15.docx {31}
P29: participant 0007.docx {23}
P30: parti-00016.docx {27}
P32: parti-8.docx {29}

Quotations
______________________________________________________________________

6:1 37 (6:6)
6:2 female (7:7)
6:3 divorced (8:8)
6:4 3 (9:9)
6:5 cannot read and write (10:10)
6:6 orthodox (11:11)
6:7 500 (In birr) (12:12)
6:8 alcohol cashier (local alcohol.. (13:13)
6:9 urban (14:14)
6:10 Four years ago (20:20)
6:11 Four years back. (22:22)
6:13 I do not fell anything; I pray.. (26:26)
6:14 I did not disclose my status t.. (28:28)
6:15 Other people who live in other.. (28:28)
6:16 I only told to my families and.. (28:28)
6:17 Now a day I am selling areki a.. (28:28)
6:18 I do not disclose my status to.. (28:28)
6:19 Being in stressful condition h.. (30:30)
6:20 ctually I was in stress even t.. (30:30)
6:21 Actually I was in stress even .. (30:30)
6:22 I was considering myself as I .. (30:30)
6:23 I was considering myself as I .. (30:30)
6:24 Polices come to our house and .. (31:31)
6:25 Social interaction between us .. (31:31)
6:26 For survival of life I take dr.. (33:33)
6:27 My family said to me do not wo.. (33:33)
6:28 Yes before corona we have no g.. (35:35)
6:30 My income was not as a former .. (40:40)
6:31 Though I fell I have no option.. (43:43)
6:32 When I heard about the serious.. (43:43)
6:33 My brothers and sisters counse.. (43:43)
6:34 They serve as by wearing [mask.. (45:45)
6:35 I do not get any assistance fr.. (38:38)
9:1 25 (6:6)
9:2 female (7:7)
9:3 married (8:8)
9:4 2 (9:9)
9:5 can read and write (10:10)
9:6 Orthodox (11:11)
9:7 4000 (In birr) (12:12)
9:8 housewife (13:13)
9:9 urban (14:14)
9:10 Three years. (17:17)
9:11 Two years (19:19)
9:13 I do not think corona though o.. (23:23)
9:14 I feel discomfort when I take .. (25:25)
9:15 there is no change in my socia.. (27:27)
9:16 My drug intake is not disrupte.. (27:27)
9:17 I did not have any support fro.. (29:29)
9:18 When corona virus emerges, mar.. (33:33)
9:19 I worried for those who live a.. (35:35)
9:20 It is difficult to say and jud.. (37:37)
9:21 I do not hear such type of inf.. (21:21)
10:1 65 (6:6)
10:2 female (7:7)
10:3 widowed (8:8)
10:4 2 (9:9)
10:5 can read and write (10:10)
10:6 600 (In birr) (12:12)
10:7 no work (13:13)
10:8 urban (14:14)
10:9 10 years. (17:17)
10:10 10 years (19:19)
10:11 have information about  severi.. (21:21)
10:12 I was afraid for only my child.. (23:23)
10:13 I am not living in a comfortab.. (25:25)
10:14 yes everybody knew before coro.. (27:27)
10:15 I am living by eating what I h.. (29:29)
10:16 But corona did not affect my l.. (29:29)
10:17 I always tell my girl to be ca.. (29:29)
10:18 I am hopeless; my life is hope.. (33:33)
10:19 “I only take my ART drug. I do.. (35:35)
10:20 there is no change in my socia.. (37:37)
10:21 Only god supports me. I am not.. (39:39)
10:24 I have no any source of income.. (41:41)
10:25 It is not stopped but I was th.. (41:41)
10:26 I said God will not bring it. .. (43:43)
10:27 Health professionals currently.. (46:46)
10:28 The previous health profession.. (46:46)
10:29 Here there was our care provid.. (46:46)
10:30 The health center was not stop.. (47:47)
10:31 Few years back we were going t.. (39:39)
11:1 40 (6:6)
11:2 female (7:7)
11:3 divorced (8:8)
11:4 2 (9:9)
11:5 can't read and write (10:10)
11:6 Orthodox (11:11)
11:7 200 (In birr) (12:12)
11:8 no work at all (13:13)
11:9 urban (14:14)
11:10 12 years (17:17)
11:11 10 years (19:19)
11:13 : I said we will die immediate.. (25:25)
11:14 Ehha ……I was in tension stress.. (27:28)
11:15 Yes I told to others as soon a.. (32:32)
11:16 Then I said if the drug is her.. (32:32)
11:17 The living condition currently.. (34:34)
11:18 When we go to fetch water it w.. (36:36)
11:19 At this time I was in anxiety .. (36:37)
11:20 Ok corona will be transmitted .. (40:41)
11:21 The one who receive money for .. (45:45)
11:22 The one who receive money for .. (45:45)
11:23 Ehhh…I am one of the poorest p.. (49:49)
11:24 My sources of income are peopl.. (49:49)
11:25 Erre…very very .previously I c.. (51:51)
11:26 “Yes everything what I told yo.. (53:53)
11:27 Yes I am receiving money from .. (55:55)
11:28 do not know …..This disease [H.. (58:58)
11:29 ” eee… my social interaction i.. (62:62)
11:30 I was taking my drug according.. (63:63)
11:31 But I am worried for the futur.. (63:63)
11:32 Corona will kill me? I do not .. (63:63)
11:33 “No I do not get any support c.. (65:65)
11:34 Never that was not specific su.. (67:67)
11:35 Metema; my place of birth was .. (69:69)
11:36 I did not get any support in t.. (71:71)
11:37 No, in the year I went to the .. (73:73)
11:38 Ehhh…I am one of the poorest p.. (75:75)
11:39 Yes I am receiving money from .. (79:80)
11:40 I was in tension stressed and .. (82:83)
11:41 Before corona health care prov.. (89:89)
11:42 After the emergency of corona .. (91:91)
11:43 No we do not insulting between.. (93:93)
11:44 I am always in tension, worrie.. (95:95)
12:1 30 (6:6)
12:2 female (7:7)
12:3 married (8:8)
12:4 4 (9:9)
12:5 orthodox (11:11)
12:6 4000 (In birr) (12:12)
12:7 housewife (13:13)
12:8 urban (14:14)
12:9 12 years (17:17)
12:10 Six years (19:19)
12:11 Interviewee: “Yes I know.” (21:21)
12:12 “ I feel as we all will die at.. (23:23)
12:13 Why do not I afraid them! Thei.. (49:49)
12:14 I afraid health professionals .. (47:48)
12:15 Ehhhhh (eyesakech) ……..when I .. (43:43)
12:16 When cold water [holy water] d.. (45:45)
12:17 Why not we think and stressed .. (45:45)
12:18 Ehhhhh (eyesakech) ……..when I .. (43:43)
12:19 As I told you earlier I am hou.. (41:41)
12:20 R:“I did not got any support f.. (39:39)
12:21 I do not disclose my status to.. (25:25)
12:22 Ok as I told to you my husband.. (31:31)
12:23 When I take the drug I remembe.. (35:35)
12:24 I am taking my ART drug every .. (37:37)
12:25 But for the future my husband .. (37:37)
16:1 42 (6:6)
16:2 male (7:7)
16:3 married (8:8)
16:4 2 (9:9)
16:5 up to grade 10 (10:10)
16:6 Orthodox (11:11)
16:7 1000 (In birr) (12:12)
16:8 barber (13:13)
16:9 10 years (17:17)
16:10 10 years (19:19)
16:11 “Yes I know. I have full infor.. (23:23)
16:12 “Now; let me tell you this inf.. (25:25)
16:14 I have full information about .. (27:27)
16:15 I have bought mask and washing.. (27:27)
16:16 Every person knew about my sta.. (29:29)
16:17 Here there is a problem on dru.. (29:29)
16:18 Before three month. There was .. (31:31)
16:19 It said if you do not receive .. (31:31)
16:20 Corona made on me to be stress.. (31:31)
16:21 Corona made me tension and dep.. (35:35)
16:22 I always use mask in my barber.. (37:37)
16:23 People said “why you were mask.. (37:37)
16:24 I have bought mask which can b.. (37:37)
16:25 I did not disclose my HIV stat.. (45:45)
16:26 Corona creates anxiety and ten.. (50:50)
16:27 These things made me to be dis.. (52:52)
16:28 “I made conflict with my wife .. (54:54)
16:29 I said you should not go to fu.. (56:57)
16:30 The society is in Bermuda by w.. (57:57)
16:31 Even I have bought her slipper.. (57:57)
16:32 . Let me return to your origin.. (57:57)
16:33 when I do this there are some .. (57:57)
16:34 people do not come to my barbe.. (57:57)
16:35 We are living in a situation w.. (59:59)
16:36 When I bought coffee from here.. (59:59)
16:37 and there is women called Enda.. (59:59)
16:38 She always goes to Bahir Dar a.. (59:59)
16:39 My work made me easily suscept.. (64:64)
16:40 Yes my neighbors and me are no.. (66:66)
16:41 The number of customers who we.. (66:66)
16:42 . I was closed my shop for abo.. (66:66)
16:43 and people assume as I was inf.. (66:66)
16:44 I come to this and contact doc.. (66:66)
16:45 Still now I am saying please d.. (66:66)
16:47 I am taking the drug correctly.. (68:68)
16:48 Lastly they give me expire dug.. (68:68)
16:49 The drug called bacterium is n.. (70:70)
16:50 I teach about corona but peopl.. (74:74)
16:51 Rather than supporting me the .. (76:76)
16:52 I closed my working place for .. (80:80)
16:53 I closed my working place for .. (80:80)
16:54 People before coronas were com.. (80:80)
16:56 I fear the drug [ ART] will be.. (90:90)
19:1 44 (6:6)
19:2 male (7:7)
19:3 married (8:8)
19:4 4 (9:9)
19:5 up to grade 7 (10:10)
19:6 Orthodox (11:11)
19:7 600 (In birr) (12:12)
19:8 farmer (13:13)
19:9 rural (14:14)
19:10 8 years (20:20)
19:11 8 years (22:22)
19:13 It was said that it affect sev.. (28:28)
19:14 Nothing I feel corona is not s.. (30:30)
19:15 No I do not afraid (32:32)
19:16 “It is being said that corona .. (34:34)
19:17 But this [corona] is not great.. (36:36)
19:18 Why I disclose my status? What.. (40:40)
19:19 Why I told I do not thing it i.. (42:42)
19:20 My life is normal in relation .. (44:44)
19:21 When corona emerges I saw new .. (52:52)
19:22 I do not spent special life in.. (54:54)
19:23 I never suspect anything that .. (58:58)
19:24 Social life is stopped previou.. (62:62)
19:25 I only took drug to prolong my.. (64:64)
19:26 I do not restrict from my soci.. (66:66)
19:27 I am living with my neighbors,.. (68:69)
19:28 For me it does not interrupt a.. (71:71)
19:29 I am taking my drug correctly (75:75)
19:30 the drug dosage form is advanc.. (77:77)
19:31 No nothing I am being supporte.. (81:81)
19:32 No my work does not relate wit.. (83:83)
19:33 No, it does not affect me. (85:85)
19:34 I do not afraid this is my nat.. (89:89)
19:35 They are treating me in a good.. (93:96)
19:36 When I told truly I consider t.. (96:96)
19:37 I am saying corona. I consider.. (98:98)
20:1 43 (6:6)
20:2 male (7:7)
20:3 married (8:8)
20:4 2 (9:9)
20:5 can read and write (10:10)
20:6 Orthodox (11:11)
20:7 200 (In birr) (12:12)
20:8 carpenter (13:13)
20:9 urban (14:14)
20:10 9 years (20:20)
20:11 7 years (22:22)
20:13 When I heard this immediately;.. (28:28)
20:14 let me tell you truly; I do no.. (30:30)
20:15 I was in Gondar when corona em.. (30:30)
20:16 But I do not feel anything at .. (30:30)
20:17 Let me tell you the truth I do.. (32:32)
20:18 If you said why; I always go t.. (36:36)
20:19 So it is necessary to be exami.. (37:37)
20:20 we said we should be examined .. (39:39)
20:21 “ok life for me …..This 40 day.. (51:51)
20:22 If you put light in the pot, i.. (51:51)
20:23 now a day the cost of goods an.. (55:55)
20:24 I did not gain rather I lost (57:57)
20:25 Ere gey) I do not suspect anyt.. (61:61)
20:26 I never stop taking ART drug. .. (65:65)
20:27 I am taking the drug appropria.. (63:63)
20:28 nothing I feel. I only come to.. (67:67)
20:29 Taking drug means to me …… (ey.. (70:70)
20:30 I am taking this drug to stay .. (72:72)
20:31 But in market I do not stay fo.. (76:76)
20:32 As soon as corona emerges she .. (78:78)
20:33 it was said that in high schoo.. (82:82)
20:34 (Endiyaw) my brother it is not.. (86:86)
20:35 I doid not get any support fro.. (88:88)
20:36 The group disintegrated becaus.. (90:90)
20:37 …corona do not speak like huma.. (118:118)
20:38 I will die ; death is not new .. (112:112)
20:39 “yebesebese zinab ayiferam” me.. (110:110)
20:40 They did not disclose themselv.. (90:91)
20:41 “Thanks my God. I am eating wi.. (95:95)
20:42 it did not brought anything on.. (98:98)
20:43 I am working in a place where .. (100:100)
20:44 “I do not afraid corona I said.. (102:102)
20:45 I told this previously they ga.. (104:105)
20:46 I was receiving health service.. (114:114)
21:1 41 (6:6)
21:2 male (7:7)
21:3 married (8:8)
21:4 3 (9:9)
21:6 4500 (In birr) (12:12)
21:7 civil servant (13:13)
21:8 urban (14:14)
21:9 8 years (20:20)
21:10 8 years. (22:22)
21:12 When I heard this for the firs.. (26:26)
21:14 I did not told my status to ot.. (28:28)
21:15 If they know, I will be discri.. (30:30)
21:16 My life related to corona is m.. (32:32)
21:17 I worried what I will do if dr.. (32:32)
21:18 I feared to come to this healt.. (32:32)
21:19 I worried what I will do if dr.. (32:32)
21:20 because what I have brought is.. (34:34)
21:21 In this corona time taking dru.. (36:36)
21:22 “In this corona time taking dr.. (36:36)
21:23 Yes, the relation with my neig.. (38:38)
21:24 For example I have a brother w.. (40:40)
21:25 “ehhhh …… no one assist me rat.. (42:42)
21:26 My wife is trying to do trade .. (44:44)
21:27 Eeeee! Different costumers com.. (46:46)
21:28 I was in tension when I hear n.. (48:48)
21:29 Ayiii….that was good but let m.. (50:50)
22:1 30 (6:6)
22:2 male (7:7)
22:3 married (8:8)
22:4 2 (9:9)
22:5 diploma (10:10)
22:6 Orthodox (11:11)
22:7 5500 (In birr) (12:12)
22:8 teacher (13:13)
22:9 urban (14:14)
22:10 2 and half years (20:20)
22:11 2 and half years (22:22)
22:13 At that time, feel worried, st.. (26:26)
22:14 Yes when we afraid I was isola.. (28:28)
22:15 I don't expose my status to ot.. (32:32)
22:16 As I tried to explain to you e.. (34:34)
22:17 I was worried when I come to t.. (36:36)
22:18 Ok when we asked still corona .. (38:38)
22:19 My relative including my wife'.. (40:41)
22:20 I am taking the drug as the pr.. (43:43)
22:21 even I interrupted drug intake.. (43:43)
22:22 Yes because when we were afrai.. (45:45)
22:23 Now medias, governments and pe.. (47:47)
22:24 People were freely moving but .. (47:47)
22:25 I did not go far away from my .. (49:49)
22:26 If one person was infected in .. (51:51)
22:27 Ok, few of them understood the.. (53:53)
22:28 Ayi …health professionals supp.. (58:58)
22:29 For me I did not face any econ.. (60:60)
22:30 but my wife was working in mar.. (60:60)
22:31 When corona come and heard inf.. (62:62)
22:32 With health care providers I h.. (64:64)
22:33 Good mean they told as what we.. (66:66)
22:34 No, if we met with the usual h.. (68:68)
22:35 Ok in relating to corona I afr.. (70:70)
22:36 Ok if corona will exist for a .. (72:72)
23:1 45 (6:6)
23:2 female (7:7)
23:3 divorced (8:8)
23:4 4 (9:9)
23:5 cannot read and write (10:10)
23:6 Orthodox (11:11)
23:7 400(In birr) (12:12)
23:8 private worker (cashier of alc.. (13:13)
23:9 urban (14:14)
23:10 7 years (20:20)
23:11 7 years (22:22)
23:13 Ehhh I feel stressed and depre.. (28:28)
23:14 Ehhaa! I have life so there is.. (30:30)
23:15 I felt only tension other thin.. (32:32)
23:16 “No I do not tell to others my.. (34:34)
23:17 I do not go to rural because I.. (45:45)
23:18 “I serve [cashiering of alcoho.. (47:47)
23:19 Yes stopping my work lead me t.. (49:49)
23:20 I said we cashiers will die pr.. (49:49)
23:21 “No because I do not live with.. (51:51)
23:22 Ok in that case it is correct .. (55:55)
23:23 Though I do not go to others, .. (59:59)
23:24 “I am taking drug appropriatel.. (65:65)
23:25 “People who move here and ther.. (63:63)
23:26 “I was serving my clients by b.. (69:69)
23:27 I do not got any special assis.. (71:71)
23:28 “I have no any relative. I am .. (73:74)
23:29 I am living in poverty I said... (76:76)
23:30 Most of the time I stop workin.. (78:78)
23:31 I told you previously. I was s.. (80:81)
24:1 55 (6:6)
24:2 male (7:7)
24:3 married (8:8)
24:4 4 (9:9)
24:5 up to grade 10 (10:10)
24:6 Orthodox (11:11)
24:7 18000 (In birr) (12:12)
24:8 Merchant (13:13)
24:9 urban (14:14)
24:10 5 years (17:17)
24:11 5 years (19:19)
24:13 I feel worried, stressed and t.. (23:23)
24:14 I was in hopeless because I al.. (23:23)
24:15 Eee … (yaw) as I told you I wa.. (25:25)
24:16 People knew about my status be.. (28:28)
24:17 People already knew about my s.. (30:30)
24:18 Before corona, I was working m.. (32:32)
24:19 when they speak about corona, .. (32:32)
24:20 “I assumed as there were many .. (34:34)
24:21 Yes I afraid corona because I .. (36:36)
24:22 First, I suspect corona will c.. (38:38)
24:23 Ok when we asked still corona .. (40:40)
24:24 People afraid I because they h.. (43:43)
24:25 Yes, my relative including my .. (42:43)
24:26 Ok yes, previously I said .you.. (52:52)
24:27 but you said previously I stop.. (51:52)
24:28 My drug intake is that I alway.. (54:54)
24:29 Ok yes, previously I said .you.. (52:52)
24:30 .but latter health professiona.. (52:52)
24:31 “People afraid me and they iso.. (56:56)
24:32 No I did not have any support .. (59:59)
24:33 Aye…corona restricts my source.. (62:62)
24:34 Aye…corona restricts my source.. (62:62)
24:35 Ayi …..what I feel is like ( b.. (64:64)
24:36 "because there is HIV/AIDS in .. (66:66)
24:37 Doctors…. They are; they are t.. (68:69)
26:1 50 (6:6)
26:2 male (7:7)
26:3 married (8:8)
26:4 6 (9:9)
26:5 up to grade 9 (10:10)
26:6 Orthodox (11:11)
26:7 1560 (In birr) (12:12)
26:8 farmer (13:13)
26:9 urban (14:14)
26:10 6 years (20:20)
26:11 6 years (22:22)
26:13 I do not feel anything before .. (26:26)
26:14 when corona emerges I already .. (28:28)
26:15 Everyone knew before corona ab.. (30:30)
26:16 But we do not get any support... (30:30)
26:17 eeeee….. I only work for eatin.. (32:32)
26:18 I spent my life by working job.. (34:34)
26:19 I suspect corona will contract.. (36:36)
26:20 No I do not face any problem r.. (38:38)
26:21 No, I do not experience such t.. (44:44)
26:22 People were afraid each other .. (45:45)
26:23 : I am attending the follow up.. (47:47)
26:24 My drug intake is that I alway.. (50:50)
26:25 I worried for others. I gave m.. (28:28)
26:26 Ayi…the normal support by safe.. (53:53)
26:27 I do not got any support. (55:55)
26:28 I was trying to produce fruits.. (59:59)
26:29 I do not feel anything. But I .. (63:63)
26:30 I think if they become ill ……i.. (65:65)
26:31 They give health information w.. (67:67)
26:32 “Ok in that side I lost my inc.. (73:73)
29:1 37 (6:6)
29:2 female (7:7)
29:3 divorced (8:8)
29:4 3 (9:9)
29:5 can't read and write (10:10)
29:6 Orthodox (11:11)
29:7 700 (In birr) (12:12)
29:8 shopkeeper (merchant) (13:13)
29:9 urban (14:14)
29:10 8 years (20:20)
29:11 8 years (22:22)
29:12 “Yes, yes, yes I know……it affe.. (24:24)
29:13 “People said to me “HIV patien.. (26:26)
29:14 where I go sir? (30:30)
29:15 if death came, I will die, I o.. (32:32)
29:16 When corona virus emerges and .. (33:33)
29:17 They [sample collectors to scr.. (33:33)
29:18 People said car drivers will i.. (33:33)
29:19 ” when I gave sample my larynx.. (37:37)
29:20 No, I am not receiving any ass.. (39:39)
29:21 No income at all especially wh.. (41:41)
29:22 I was worried, stressed and in.. (43:43)
29:23 They were saying on us “be far.. (45:45)
30:1 40 (6:6)
30:2 married (8:8)
30:3 5 (9:9)
30:4 up to grade 11 (10:10)
30:5 Orthodox (11:11)
30:6 5000 (In birr) (12:12)
30:7 merchant (13:13)
30:8 urban (14:14)
30:9 11 years (20:20)
30:10 8 years (22:22)
30:11 “Yes I know. The message was d.. (24:24)
30:12 I feared and was in tension as.. (26:26)
30:13 they come to my shop to buy di.. (26:26)
30:14 I said sorry and was in worrie.. (28:28)
30:15 Yes, I closed my shop for few .. (30:30)
30:16 I did not told my status to ot.. (32:32)
30:17 Because I think if I told my s.. (34:34)
30:18 As I told my source of income .. (36:36)
30:19 Yes many people come to my sho.. (38:38)
30:20 People consider me as I am inf.. (40:40)
30:21 I remember when I swallow my [.. (42:42)
30:22 I remember when I swallow my [.. (42:42)
30:23 I always take my drug appropri.. (44:44)
30:24 I teach about corona but peopl.. (47:47)
30:25 I did not got any special supp.. (50:50)
30:26 As I told before corona I was .. (57:57)
30:27 Now, as I told earlier, I was .. (59:59)
32:1 30 (6:6)
32:2 female (7:7)
32:3 married (8:8)
32:4 4 (9:9)
32:5 grade 10 completed (10:10)
32:6 Orthodox (11:11)
32:7 2000 (In birr) (12:12)
32:8 farmer (13:13)
32:9 urban (14:14)
32:10 4 years (20:20)
32:11 4 years (22:22)
32:12 “When corona comes, I afraid b.. (28:28)
32:13 “I felt anxiety when I head as.. (30:30)
32:14 “Because the drug will be inte.. (34:34)
32:15 I spend my life by taking ART .. (38:38)
32:16 No I have no anything what I s.. (42:42)
32:17 Because I should take care my .. (40:40)
32:18 Yes, because they said “do not.. (52:53)
32:19 I was taking coming and collec.. (61:61)
32:20 People from rural afraid and d.. (64:64)
32:21 No I only support myself I hav.. (68:68)
32:22 Eeeee. Ok on that side the inc.. (74:74)
32:23 Because in last march school w.. (76:76)
32:24 Eeee, from farming (eyesakech).. (78:78)
32:25 “I was stressed because corona.. (81:81)
32:26 “Second; when this happen [whe.. (83:83)
32:27 Yes it was distributed in medi.. (85:85)
32:28 Eeeee why we afraid them rathe.. (87:87)
32:29 I do not experience anything t.. (95:95)

Codes
______________________________________________________________________

acceptane on the severity of COVID-19=not accepted {4-0}
afread health professionals {3-0}
age {15-0}
anxiety,worried and tenssion {3-1}
asking financial support {1-0}
cause for decreasment of  social interaction {1-0}
conditions how HIV aquired {1-0}
cuase for income  decreasment {2-1}
cuase of not disclosing {1-0}
cuase of relief from stress, worried {1-0}
decrease household income {13-1}
decrease income {5-0}
diffecult life condition {6-0}
difficult to express negative or positive social relation {1-0}
disclosed their status {8-0}
disclosure status {0-0}
do not have information about severity of COVID-19 {1-0}
no problem of drug intake {3-0}
drug intake not intrupted {19-0}
after intrrupted of ART drug intake continued {2-0}
economic =source of income {0-0}
economic experience {0-2}
economicimpact of covid-19 {3-0}
educational status {13-0}
educational status=did not attend {1-0}
fear of contracting COVID-19 {0-0}
financial conditions after emergency of covid=dicrease {0-0}
good social interaction {3-0}
got family assistance {4-0}
has/have not worked {1-1}
have information about severity of COVID-19 {1-0}
health care providers not treat them apppropriatly {5-0}
immediatly feeling at time of dx of HIV {0-0}
increase costs {7-0}
intrrupted ART drug intake {9-1}
isolated by others {2-0}
life spend =no difference from pre-COVID-19 {2-0}
marital status {15-0}
monthly income {16-0}
no changd in social interaction {12-0}
no change in household income {5-0}
no change in social relation {1-0}
no feeling for themselves, worried for others {11-1}
no feeling in relating COVID-19-during ART drug intake {1-0}
no financial support {17-0}
no life change related to COVID-19 {1-0}
not disclosed {2-0}
not fear {10-0}
not got  any support {5-0}
not got any assistanec  from community and government {6-0}
not got psychological support {1-0}
number of families {16-0}
occupational status {16-0}
only disclosed to family {5-0}
poor service quality {5-0}
prevention mechanism of COVID-19 {1-0}
cause of stress {1-0}
psycological effct of COVID-19=tension {0-0}
psycological effct of COVID-19=tenssion {0-0}
psycological feeling due to social interaction {0-0}
reason for not disclosing {6-0}
reason for not keeping social distance {0-0}
religion {16-0}
residence {13-0}
response to tension {9-0}
response to tenssion {2-0}
risk  of contracting COVID-19 experienced by people on ART {17-0}
sex {17-0}
smooth  interaction with health care providers {12-0}
social interactio reduced {7-0}
social interaction =not changed {0-0}
social interaction decreased {24-0}
social interaction gradually become smooth {1-0}
social interaction=difficult to express negative or positive social {0-0}
source of income {2-0}
stress about future drug availabity {0-0}
support=no financial support {2-0}
tenssion {1-0}
uncertain about future economic condition {1-0}
uncertain for future ART drug availability {10-0}
worrid, anxiety and tenssion {51-0}
worried for others not for themselves {0-0}
years in ART {16-0}
years living with HIV {16-0}

Primary Doc Families
______________________________________________________________________

female (0)
male (0)

Code Families
______________________________________________________________________

assistance (5)
drug intake (4)
economic impact (13)
psycological experience (17)
social impact (8)

Code-Links
______________________________________________________________________
