# Supplementary material for: Lived experience of people on anti-retro viral therapy in the context of covid-19: A phenomenological study
Source: PLoS One. 2023 Nov 2;18(11):e0286292. doi: 10.1371/journal.pone.0286292 (PMC10621913; doi:10.1371/journal.pone.0286292)
Supplement: Attachment — Submitted filename: Rebuttal letter.docx [file pone.0286292.s002.docx]

Date 19/12/2022	Rebuttal letter

**Dear editor**

**Plosone journal editor**

We express our cordial gratitude for the valuable constructive reviews of our paper entitled with “the lived experience of people on ART in context of COVID-19 in Tach Gayint district a phenomenological study”. The reviewer’s comments have helped us to further strengthen the overall quality of the paper. With great care, we have incorporated all the suggestions/corrections as proposed by the reviewer. The specific responses to the reviewer’s comment are listed below.

Comment 1: The purpose of the study was not well-expressed under the introduction.

Response: The manuscript addressed this issue and updated.

Comment 2: The authors did not summarize the demographics of the participants appropriately.

Response: it is very important concept and we saw it and summarized.

Comment 3: with labeling and ambiguity with usage of words. Participant 12 is labeled as Me instead of M. Educational status is tagged as degree. What kind of degree? Bachelors’, Master’s or a PhD, or are they lumped together?

Response; it is very constructive comment and we updated it in the paper

Comment 4: The authors did not compare their results properly with was done elsewhere.

Response: we saw it detail and it is updated.

Comment 5: Under the limitation, the authors did account for what they do with "shy people" who could not completely share their experiences. Secondly the authors said rural residents were not accessed. One of the participants coded P9, was accessed and from the rural area. The author could have written that they couldn't access enough participants from rural areas rather than saying that they were not accessed, or is that the status 'rural' for that client was an error or an interpolation?

Response we admired giving such type of constructive comment and we incorporated the issue given.

Comment 6: The authors did not make any recommendations based on their findings.

Response: we revised it and tried to match the result with recommendation

Sincerely
