# Supplementary material for: Lived experience of people on anti-retro viral therapy in the context of covid-19: A phenomenological study
Source: PLoS One. 2023 Nov 2;18(11):e0286292. doi: 10.1371/journal.pone.0286292 (PMC10621913; doi:10.1371/journal.pone.0286292)
Supplement: Attachment — Submitted filename: response to reviewer.docx [file pone.0286292.s003.docx]

Date 25/3/2023	Rebuttal letter

**Dear editor**

**Plosone journal editor**

We express our cordial gratitude for the valuable constructive reviews of our paper entitled with “the lived experience of people on ART in context of COVID-19 in Tach Gayint district a phenomenological study”. The reviewer’s comments have helped us to further strengthen the overall quality of the paper. With great care, we have incorporated all the suggestions/corrections as proposed by the reviewer. The specific responses to the reviewer’s comment are listed below.

Comment 1: The researchers did not provide good detailed information about the study design.

Response: The manuscript addressed this issue and updated.

Comment 2: It is important to describe the participants in a separate part, omitting the sample size part.

Response: it is very important concept and we saw it and described.

Comment 3: Deletion of the name (method and procedures of data collection) and amending it to (the context of the study).

Response; it is very constructive comment and we updated it in the paper

Comment 4: There is an overlap between the elements of the paper (a note that needs great attention)

Response: we saw it detail tried to delete the overlapped one.

Comment 5: The procedures of credibility and reliability are not clear, presented in a narration without linking to the study

Response: the issues of credibility and reliability are revised again and are linked to the study

Comment 6: The steps of qualitative analysis presented superficially are not sufficient and the presentation of result require focus

Response: we admired giving such type of constructive comment and we incorporated the issue given.

Comment 7: Add study limitations and future studies

Response: we revised it and added future studies and other limitations.

Sincerely
